# Long-acting injectable antipsychotic (LAI) prescribing trends during COVID-19 restrictions in Canada: a retrospective observational study

**DOI:** 10.1186/s12888-021-03646-9

**Published:** 2021-12-20

**Authors:** Kyle A. McKee, Candice E. Crocker, Philip G. Tibbo

**Affiliations:** 1 Department of Psychiatry, Dalhousie University, 5909 Veterans Memorial Lane, Halifax, Nova Scotia B3H 2E2 Canada; 2 Department of Diagnostic Radiology, Dalhousie University, Halifax, Nova Scotia Canada

**Keywords:** COVID-19, Long-acting injectable antipsychotics, Schizophrenia, Psychotic disorders, Prescribing data

## Abstract

**Background:**

The COVID-19 pandemic has had significant impacts on how mental health services are delivered to patients throughout Canada. The reduction of in-person healthcare services have created unique challenges for individuals with psychotic disorders that require regular clinic visits to administer and monitor long-acting injectable antipsychotic medications.

**Methods:**

To better understand how LAI usage was impacted, national and provincial patient-level longitudinal prescribing data from Canadian retail pharmacies were used to examine LAI prescribing practices during the pandemic. Prescribing data on new starts of medication, discontinuations of medications, switches between medications, antipsychotic name, concomitant medications, payer plan, gender and age were collected from January 2019 to December 2020 for individuals ≥18-years of age, and examined by month, as well as by distinct pandemic related epochs characterized by varying degrees of public awareness, incidence of COVID-19 infections and public health restrictions.

**Results:**

National, and provincial level data revealed that rates of LAI prescribing including new starts, discontinuations and switches between LAI products remained highly stable (i.e., no statistically significant differences) throughout the study period.

**Conclusions:**

Equal numbers of LAI new starts and discontinuations prior to and during the pandemic suggests prescribing of LAI antipsychotics, for those already in care, continued unchanged throughout the pandemic. The observed consistency of LAI prescribing contrasts with other areas of healthcare, such as cardiovascular and diabetes care, which experienced decreases in medication prescribing during the COVID-19 pandemic.

**Supplementary Information:**

The online version contains supplementary material available at 10.1186/s12888-021-03646-9.

## Background

### COVID-19 in Canada and service delivery

In response to the COVID-19 pandemic (severe acute respiratory syndrome coronavirus 2 [SARS-CoV-2] and emerging variants), most of Canada (and the world) adopted quarantine measures. These measures ranged from stay-at-home guidelines, to varying degrees of restricted movement and enforced business closures. COVID-19 has uniquely impacted those more vulnerable in society due to mental health concerns such as schizophrenia and related psychotic disorders, as well as the clinical teams who work to provide stability for these patients. During the pandemic, mental health services had to change in response to increasing levels of confinement and the related demands of being asked to work from home, including restricting face-to-face healthcare visits and opting for more telephone or video platform interviews with patients. The types of restrictions adopted by particular healthcare providers varied widely from province-to-province (and within provinces by health authority). As well, restrictions were regularly revised within regions as case numbers and hospitalizations fluctuated.

However, individuals with a psychotic disorder who were on long-acting injectable antipsychotic (LAI) therapy still needed to receive their medication, and thus service delivery had to pivot to align with the public health requirements imposed by local health authorities. In Canada, LAI injections need to be performed by a trained professional (e.g., psychiatrist, general practitioner, registered nurse, nurse practitioner), and are typically administered within psychiatric departments (inpatient/outpatient), mental health clinics (hospital/community), or while visiting with a general practitioner. While COVID-related restrictions were not uniform across these settings they typically included more home visits for delivery of LAIs, more restricted times to attend clinics to receive LAIs, limits on who (if anyone) could accompany a patient while visiting a clinic, potentially less patient interaction with clinical teams, and more patients receiving their LAI from an unfamiliar provider. Broader challenges faced by individuals with schizophrenia and related psychoses during the COVID-19 pandemic have been recently reviewed [1–3].

Both the Canadian Psychiatric Association (CPA) and American Psychiatric Association (APA) issued guidelines urging hospitals, clinics, and other facilities to continue treatment with LAIs, as it was their view that LAI treatment should be considered a medically necessary procedure [4, 5]. Whereby, the risks associated with discontinuation of LAI medication for patients (e.g., relapse related to inadequate oral medication adherence) were seen as greater than the potential risk of exposure/spread of coronavirus related to continued clinic/home visits to monitor LAI administration. However, the literature shows there were a mixture of reactions to the restrictions imposed by the pandemic in terms of LAI prescribing practices. Some jurisdictions sided with the recommendations from the CPA and APA, choosing to continue LAI administration [3, 6], or even considered switching to compounds with longer half-life [7–9] - whereas others took the position that patients should be switched to oral medication due fears that some patients would refuse to come into the clinic, or that patients with lower socioeconomic status (SES) would have reduced access to public transportation, thereby limiting their access to clinics [10]. Some clinics also expressed fears related to limited access to LAI medications due to restrictions, or cancellations of medical exports [11]. It is unclear however what the overall impact of these various approaches has been on LAI prescribing and delivery during the pandemic.

This study aimed to fill gaps in knowledge regarding delivery of mental health care during a pandemic by examining LAI prescribing practices during unique time frames prior to and during the pandemic. While there is some limited research on mental health effects of prolonged quarantine on overall mental health status of populations [12–14], there does not appear to be any Canadian data on changes in prescribing practices during the pandemic; specifically, changes in LAI prescribing in schizophrenia and related psychoses. We do not know if during the pandemic LAI use was increased or decreased due to the changes in healthcare delivery and public health restrictions. Of particular interest is whether there has been change in the use of the three-monthly LAI formulation – an arguably advantageous method of medication delivery during times of diminished/changed face-to face interaction between patients and their clinical teams. Here, we examined the approach to mental health care during the pandemic in the Canadian context by examining LAI prescribing practices at a national and also at a provincial level to address some variation in COVID-19 restrictions regionally.

## Methods

### Data source

This retrospective observational study examined national and provincial patient-level longitudinal prescribing data from Canadian retail pharmacies from January 2019 through to December 2020, using the IQVIA Canadian Longitudinal Prescription (LRx) database [15]. This database includes anonymized, longitudinal, patient level information on approximately 72% of national pharmacy prescriptions in Canada. The second-generation long-acting injectable antipsychotic (SG-LAI) products captured by this dataset included Paliperidone palmitate (Invega Sustenna, and Invega Trinza), Risperidone (Risperdal Consta), Aripiprazole (Abilify Maintena), and first-generation long-acting injectable antipsychotics (FG-LAI) including Zuclopenthixol (Clopixol depot), Flupentixol (Fluanxol depot), Fluphenazine (Modecate depot), Haloperidol (Haloperidal LA), Pipotiazine (Piportil depot), and all relevant generics. The dataset also captured the oral second-generation antipsychotics (SGAs) Aripiprazole (Abilify), Clozapine (Clozaril), Paliperidone palmitate (Invega), Lurasidone HCl (Latuda), Brexpiprazole (Rexulti), Risperidone (Risperdal), Risperidone (Risperdal M), Asenapine (Saphris), Quetiapine (Seroquel), Ziprasidone (Zeldox), Olanzapine (Zyprexa), and related generics. Oral first-generation antipsychotics (FGA) of interest included Phenothiazines [e.g., Fluphenazine (Modecate)], as well as other oral FGAs [e.g., Loxopine (Loxapac)], and related generic formulations. The data was collected from retail pharmacies across Canada and consequently captures prescription information from across Canada. A known limitation of the chosen database includes the inability to track patients that switch between pharmacies (i.e., travelling patients). As such, patients switching pharmacies appear as unique patients. To address this limitation, patients included in this study must have had at least 30-days of data history with a pharmacy. Furthermore, patient data was excluded if age was unknown, age < 18 years, or if gender was unknown.

The database collects the payer information (including private drug plan, public drug plan, or cash transaction), drug product information (drug identification number, trade name, chemical name, formulation, and dosage), and basic demographic information (age, gender, and geographic location). The database does not include information on whether the LAI medication was administered or what the clinical indication for the prescription was, only whether the prescription was filled. No personally identifying data was included in this dataset. A cell suppression threshold value of 5 was used to further protect the confidentiality of patient data. Consequently, some provincial level data was combined and is presented more generally as Atlantic/Western Canada.

### Study design

**Fig. 1:**
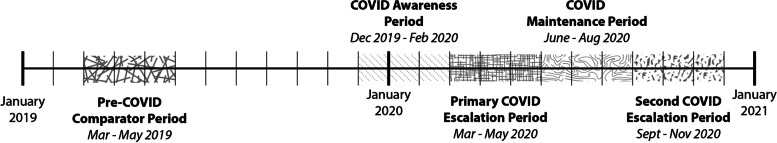
Study timeline, including epochs of interest. LAI new starts, LAI discontinuations and PP1 to PP3 switches were examined by month from January 2019 to December 2020. LAI, long-acting injectable antipsychotic; PP1, one-month paliperidone palmitate; PP3, three-month paliperidone palmitate

Using a retrospective design, data were collected from January 2019 to December 2020 for individuals 18-years and older. Data was examined by month, and by the following time epochs during the COVID-19 pandemic (see Fig. 1, Study Timeline).

(i) Pre-COVID comparator period (March – May 2019); a three-month period in the previous year aligning with Canada’s initial COVID escalation period.

(ii) COVID awareness/sensitization period (December 2019–February 2020); a period in which the virus was known to be active in the country, news reports were publicizing the emerging pandemic, but few preventative measures were yet being taken.

(iii) primary COVID escalation period (March – May 2020); the virus was active and being locally transmitted in all Canadian provinces, and states of emergency were active in all provinces.

(iv) COVID maintenance period (June – August 2020); a period where initial transmission rates had stabilized, but preventative measures remained active in most communities.

(v) second COVID escalation period (September – November 2020); a period where transmission rates began to increase as the country entered the second wave of the pandemic. Second wave infections peaked during January 2021 [16].

### Outcomes

The primary outcome of this retrospective study was the number of LAI new starts (i.e., occurrence of an LAI prescription, with no prior LAI prescription in the past 30-days), during Canada’s COVID-19 response; December 2019 to November 2020. The number of LAI discontinuations (i.e., discontinuation of an LAI prescription with no subsequent LAI refills within 30-days of the medication ending) were also evaluated to examine whether oral antipsychotics may have been resumed in the later COVID epochs. While it is typical for a patient to refill before, or on, the date their current medication supply ends, a 30-day buffer was used to account for patients that may have delayed their refill. Switches between a one-month paliperidone palmitate formulation (PP1; Invega Sustenna, Janssen Pharmaceuticals, Inc.) and a three-month paliperidone palmitate formulation (PP3; Invega Trinza, Janssen Pharmaceuticals, Inc.) were also examined. Invega Trinza was the only LAI three-month formulation approved for use in Canada during the study period. Switches were defined as a new paliperidone palmitate LAI prescription (either PP1, or PP3) that were dispensed within 30-days of the previous paliperidone palmitate product prescription ending. Note that LAI switching in this context only includes switching between LAI formulations of paliperidone palmitate, and not patients who discontinued and then started again.

The secondary aims of this study were to examine the above data with respect to patient demographics (i.e., age, gender), possible provincial differences in prescribing, payer type (i.e., public, private, other), as well as concomitant neuroleptic medications (including SSRIs/SNRIs, tricyclic antidepressants, sedative hypnotics, benzodiazepines, anti-epileptic, and other mood stabilizing medications).

### Data analysis

Descriptive statistics were the focus of analysis in this retrospective cohort study. Comparisons between time epochs were employed by chi-squared tests to examine potential differences in the average monthly proportion of LAI prescription new starts, discontinuations, and switches between time epochs. Following the methodology and analysis plan outlined, IQVIA conducted the patient level analyses (validated by the study lead).

## Results

Population demographics for LAI new starts and discontinuations are shown in Table 1. Demographics were reported by unique patients as of their first LAI new start, or LAI discontinuation. The LAI prescription (LAI Rx) patients were more likely to be men (64.5% of new starts, and 64.9% of discontinuations), more likely to be younger (e.g., 45.6% for new starts, and 43.0% discontinuing patients were between 18- and 35-years of age), and most LAI Rx’s were paid by publicly covered insurance plans (80.4% for new starts, and 82.1% for discontinuing patients). Demographics for patients switching between one-month and three-month formulations of paliperidone palmitate LAIs (from one-month to three-month, and from three-month to one-month) are reported in Table 2.

**Table 1:** Population demographics including unique patient LAI new starts and LAI discontinuations; stratified by gender, age, province/region, and payer type. Reported as total number of events (n) and percentage (%)

**LAI New Starts: January 2019 to December 2020**		**Number of Unique Patients by Epoch**				
**Characteristic**	**All LAI New Starts** **(*n*** **= 26,770)**	**COVID Awareness Period** **(*n*** **= 4459)**	**Primary COVID Escalation Period** **(*n*** **= 4518)**	**COVID Maintenance Period** **(*n*** **= 4500)**	**Second COVID Escalation Period** **(*n*** **= 4434)**	**Pre-COVID Comparator Period** **(*n*** **= 4303)**
Gender						
Women, n (%)	9504 (35.5)	1535 (34.4)	1636 (36.2)	1599 (35.5)	1560 (35.2)	1504 (35.0)
Men, n (%)	17,266 (64.5)	2924 (65.6)	2882 (63.8)	2901 (64.5)	2874 (64.8)	2799 (65.0)
Age In Years						
18–35, n (%)	12,205 (45.6)	2073 (46.5)	2122 (47.0)	2101 (46.7)	2205 (49.7)	1890 (43.9)
36–50, n (%)	8121 (30.3)	1334 (29.9)	1338 (29.6)	1370 (30.4)	1284 (29.0)	1369 (31.8)
51–64, n (%)	4444 (16.6)	709 (15.9)	762 (16.9)	734 (16.3)	665 (15.0)	723 (16.8)
65+, n (%)	2000 (7.5)	343 (7.7)	296 (6.6)	295 (6.6)	280 (6.3)	321 (7.5)
Province						
AB, n (%)	2410 (9.0)	381 (8.5)	439 (9.7)	422 (9.4)	413 (9.3)	380 (8.8)
Atlantic CA, n (%)	1983 (7.4)	350 (7.8)	324 (7.2)	287 (6.4)	317 (7.1)	308 (7.2)
BC, n (%)	2636 (9.8)	424 (9.5)	436 (9.7)	441 (9.8)	418 (9.4)	413 (9.6)
MB, n (%)	537 (2.0)	97 (2.2)	88 (1.9)	98 (2.2)	93 (2.1)	104 (2.4)
ON, n (%)	8214 (30.7)	1389 (31.2)	1428 (31.6)	1414 (31.4)	1410 (31.8)	1347 (31.3)
QC, n (%)	10,166 (38.0)	1657 (37.2)	1669 (36.9)	1708 (38.0)	1651 (37.2)	1620 (37.6)
SK, n (%)	824 (3.1)	161 (3.6)	134 (3.0)	130 (2.9)	132 (3.0)	131 (3.0)
Payer						
Cash / Unknown source, n (%)	1623 (6.1)	240 (5.4)	251 (5.6)	276 (6.1)	308 (6.9)	215 (5.0)
Government, n (%)	21,528 (80.4)	3550 (79.6)	3619 (80.1)	3619 (80.4)	3550 (80.1)	3563 (82.8)
Private, n (%)	3619 (13.5)	669 (15.0)	648 (14.3)	605 (13.4)	576 (13.0)	525 (12.2)
**LAI Discontinuations: January 2019 to December 2020**		**Number of Unique Patients, by Epoch**				
**Characteristic**	**All LAI Discontinuations** **(*n*** **= 26,548)**	**COVID Awareness Period** **(*n*** **= 4242)**	**Primary COVID Escalation Period** **(*n*** **= 4507)**	**COVID Maintenance Period** **(*n*** **= 4382)**	**Second COVID Escalation Period** **(*n*** **= 4509)**	**Pre-COVID Comparator Period** **(*n*** **= 4347)**
Gender						
Women, n (%)	9331 (35.1)	1462 (34.5)	1575 (34.9)	1604 (36.6)	1546 (34.3)	1549 (35.6)
Men, n (%)	17,217 (64.9)	2780 (65.5)	2932 (65.1)	2778 (63.4)	2963 (65.7)	2798 (64.4)
Age In Years						
18–35, n (%)	11,411 (43.0)	1851 (43.6)	1915 (42.5)	2041 (46.6)	2087 (46.3)	1879 (43.2)
36–50, n (%)	8124 (30.6)	1293 (30.5)	1399 (31.0)	1301 (29.7)	1342 (29.8)	1340 (30.8)
51–64, n (%)	4714 (17.8)	718 (16.9)	815 (18.1)	737 (16.8)	711 (15.8)	746 (17.2)
65+, n (%)	2299 (8.7)	380 (9.0)	378 (8.4)	303 (6.9)	369 (8.2)	382 (8.8)
Province						
AB, n (%)	2457 (9.3)	393 (9.3)	398 (8.8)	425 (9.7)	394 (8.7)	442 (10.2)
Atlantic CA, n (%)	1784 (6.7)	286 (6.7)	284 (6.3)	272 (6.2)	300 (6.7)	295 (6.8)
BC, n (%)	2721 (10.2)	390 (9.2)	529 (11.7)	452 (10.3)	418 (9.3)	476 (11.0)
MB, n (%)	556 (2.1)	92 (2.2)	126 (2.8)	105 (2.4)	92 (2.0)	110 (2.5)
ON, n (%)	8242 (31.0)	1342 (31.6)	1456 (32.3)	1404 (32.0)	1445 (32.0)	1338 (30.8)
QC, n (%)	10,003 (37.7)	1610 (38.0)	1594 (35.4)	1579 (36.0)	1716 (38.1)	1559 (35.9)
SK, n (%)	785 (3.0)	129 (3.0)	120 (2.7)	145 (3.3)	144 (3.2)	127 (2.9)
Payer						
Cash / Unknown source, n (%)	1487 (5.6)	237 (5.6)	227 (5.0)	262 (6.0)	264 (5.9)	219 (5.0)
Government, n (%)	21,785 (82.1)	3468 (81.8)	3716 (82.4)	3549 (81.0)	3656 (81.1)	3602 (82.9)
Private, n (%)	3276 (12.3)	537 (12.7)	564 (12.5)	571 (13.0)	589 (13.1)	526 (12.1)

To protect sensitive personal information, small cell sizes of 1–5 subjects were managed by grouping or excluding specific categories. *NB, NL, NS and PE have been combined into Atlantic Canada. The unique patient demographics are based on the earliest new start or discontinuation included in this study or in an epoch. LAI, long-acting injectable antipsychotic; AB, Alberta; BC, British Columbia; MB, Manitoba; ON, Ontario; QC, Quebec; SK, Saskatchewan

**Table 2:** Population demographics including unique patient LAI switch events; stratified by gender, and age. Reported as total number of events (n) and percentage (%)

**One-Month Paliperidone Palmitate (PP1) to Three-Month Paliperidone Palmitate (PP3) Switches**						
**January 2019 to December 2020**		**Number of Unique Patients, by Epoch**				
**Characteristic**	**All PP1 to PP3 Switches** **(*n*** **= 2770)**	**COVID Awareness Period** **(*n*** **= 308)**	**Primary COVID Escalation Period** **(*n*** **= 420)**	**COVID Maintenance Period** **(*n*** **= 265)**	**Second COVID Escalation Period** **(*n*** **= 301)**	**Pre-COVID Comparator Period** **(*n*** **= 442)**
Gender						
Women, n (%)	822 (29.7)	86 (27.9)	119 (28.3)	80 (30.2)	87 (28.9)	149 (33.7)
Men, n (%)	1948 (70.3)	222 (72.1)	301 (71.7)	185 (69.8)	214 (71.1)	293 (66.3)
Age In Years						
18–35, n (%)	1002 (36.2)	102 (33.1)	150 (35.7)	113 (42.6)	129 (42.9)	149 (33.7)
36–50, n (%)	922 (33.3)	104 (33.8)	133 (31.7)	81 (30.6)	92 (30.6)	158 (35.7)
51–64, n (%)	604 (21.8)	79 (25.6)	86 (20.5)	50 (18.9)	59 (19.6)	102 (23.1)
65+, n (%)	242 (8.7)	23 (7.5)	51 (12.1)	21 (7.9)	21 (7.0)	33 (7.5)
**Three-Month Paliperidone Palmitate (PP3) to One-Month Paliperidone Palmitate (PP1) Switches**						
**January 2019 to December 2020**		**Number of Unique Patients, by Epoch**				
**Characteristic**	**All PP3 to PP1 Switches** **(*n*** **= 433)**	**COVID Awareness Period** **(*n*** **= 64)**	**Primary COVID Escalation Period** **(*n*** **= 48)**	**COVID Maintenance Period** **(*n*** **= 56)**	**Second COVID Escalation Period** **(*n*** **= 59)**	**Pre-COVID Comparator Period** **(*n*** **= 55)**
Gender						
Women, n (%)	133 (30.7)	22 (34.4)	18 (37.5)	15 (26.8)	18 (30.5)	18 (32.7)
Men, n (%)	300 (69.3)	42 (65.6)	30 (62.5)	41 (73.2)	41 (69.5)	37 (67.3)
Age In Years						
18–35, n (%)	152 (35.1)	23 (35.9)	17 (35.4)	17 (30.4)	21 (35.6)	23 (41.8)
36–50, n (%)	151 (34.9)	22 (34.4)	21 (43.8)	17 (30.4)	18 (30.5)	17 (30.9)
51+, n (%)	130 (30.0)	19 (29.7)	10 (20.8)	22 (39.3)	20 (33.9)	15 (27.3)

The unique patient demographics are based on the earliest switch event included in this study or in an epoch. LAI, long-acting injectable antipsychotic

**Fig. 2:**
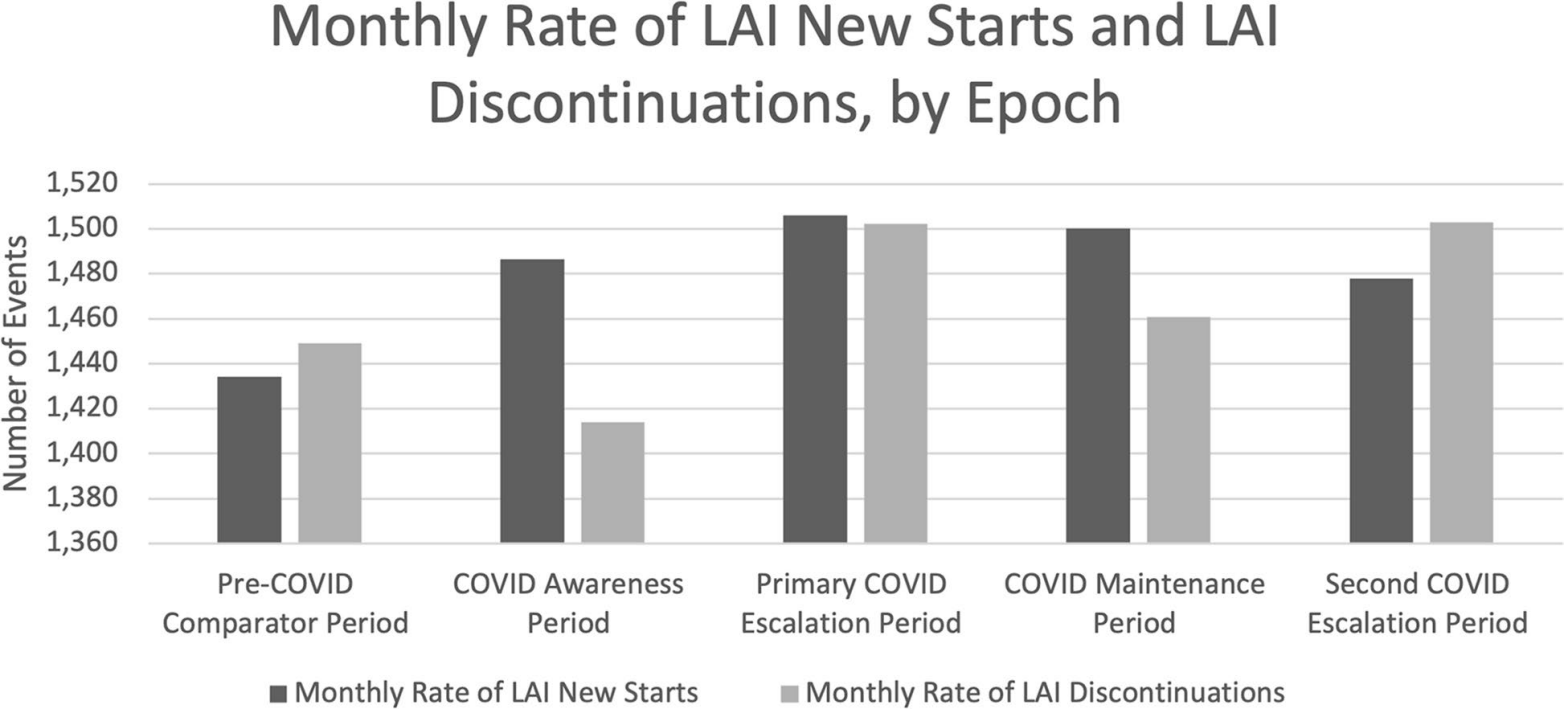
Average monthly rates of LAI new starts and LAI discontinuations, by epoch for national data. The numbers of new starts and discontinuations of LAI prescriptions are shown for nationwide data. These data reflect the number of unique patients based on the earliest switch event included in the study timeline, or in an epoch. Patients may have multiple switch events in the study or in a given epoch but are only counted once for this figure. LAI, long-acting injectable antipsychotic

**Fig. 3:**
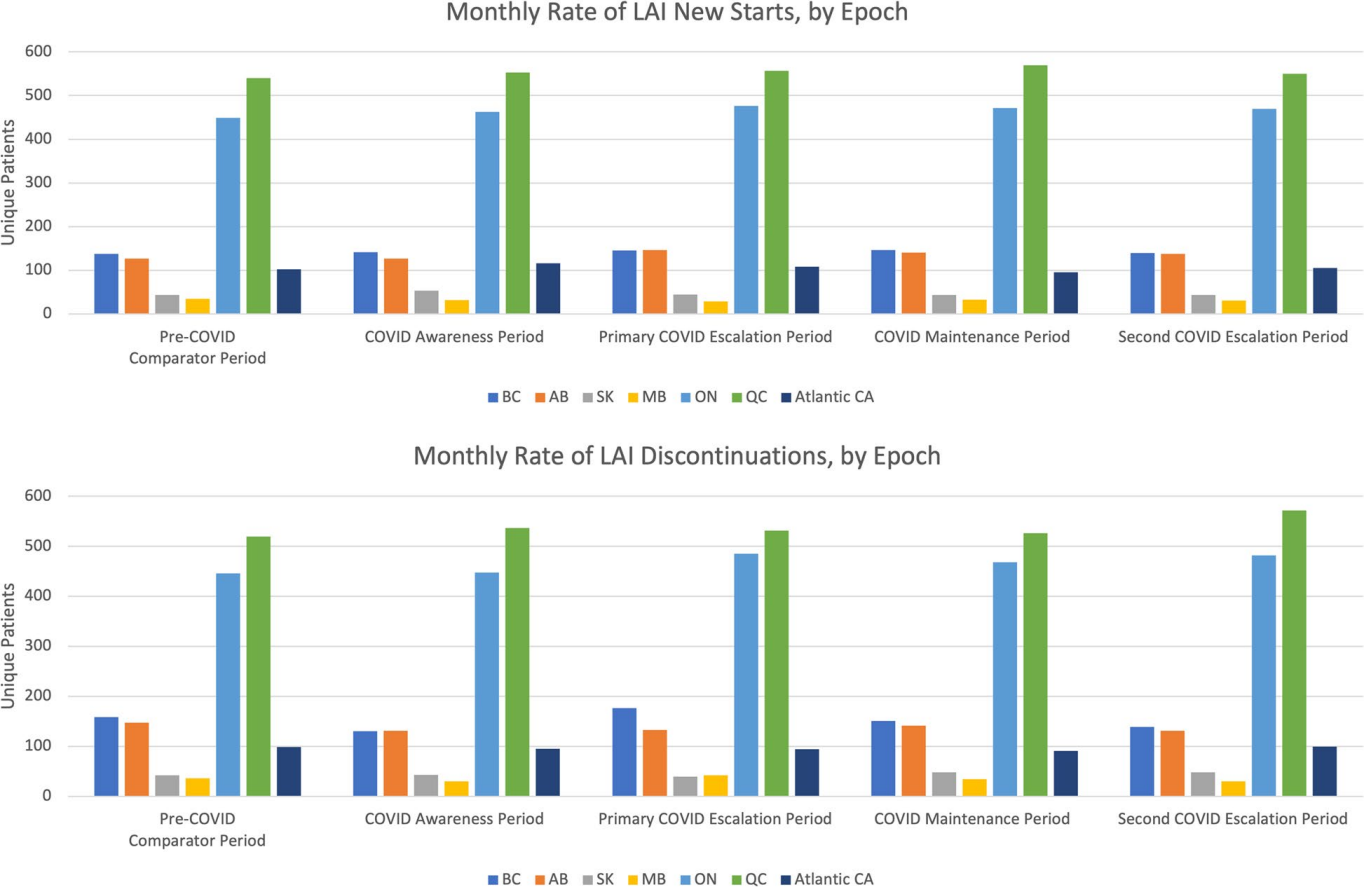
Average monthly rates of LAI new starts and LAI discontinuations, by epoch for Provincial/regional data. *Note.* Due to low values, to maintain patient confidentiality, data from New Brunswick, Nova Scotia, Newfoundland and Prince Edward Island were combined into the category ‘Atlantic Canada’*.* Data reflects the number of unique patients based on the earliest switch event included in the study timeline, or in an epoch. Patients may have multiple switch events in the study or in a given epoch but are only counted once for this figure. LAI, long-acting injectable antipsychotic; AB, Alberta; BC, British Columbia; MB, Manitoba; ON, Ontario; QC, Quebec; SK, Saskatchewan

There was no impact of the COVID-19 pandemic on LAI Rx rate at the national level. The proportion of discontinuations and new starts were not significantly different over any of the epochs examined (Fig. 2). See supplementary Table 1 for Chi-square results. As well, rates of LAI new starts and discontinuations remained stable (i.e., not statistically different) across epochs when data were examined at the provincial level (Fig. 3). Over half of the new starts and discontinuations were in Ontario and Quebec.

Overall, LAI Rx new starts were 96.3% for SG-LAIs, and 3.7% FG-LAIs. When specific formulations of SGAs were examined, paliperidone palmitate was the most commonly prescribed (46.9%), followed closely by aripiprazole (42%). Use of anti-epileptics was the most frequent concomitant medication class prescribed, with 20.5% of new LAI Rx starts also having an anti-epileptic drug dispensed. Within this concomitant medication class, 45.3% of the prescriptions were for valproic acid and 7.1% were for lamotrigine.

In the context of restricted access to healthcare during the pandemic, using a three-month formulation could be envisioned as a more advantageous strategy compared with a one-month formulation, as it would allow for less frequent clinic or pharmacy visits. For this reason, we additionally examined switches from one-month to three-month LAI antipsychotic formulations. There were 2770 switches from one-month paliperidone palmitate LAI formulations to three-month paliperidone palmitate formulation (global study period January 2019, to December 2020). Only 433 individuals switched from three-month to one-month formulations over this same global study period. When the epochs of the study are considered, all epochs had significantly more switches to the three-month formulation (accounting for 82.8% of all switches during the COVID awareness period; 89.7% during the primary COVID escalation period; 82.6% during COVID maintenance period; and 83.6% during the second COVID escalation period). Chi-square analysis (Supplementary Table 2) showed that the proportion of average monthly one-month paliperidone palmitate LAI formulations to three-month paliperidone palmitate switches during the primary COVID escalation period were significantly greater than the COVID maintenance period (*p* < 0.01), and second COVID escalation period (*p* < 0.01). However, contrary to our hypothesis, the pre-COVID comparator period had the overall highest proportion of average monthly switches between one-month paliperidone palmitate LAI formulations to three-month paliperidone palmitate and was shown to have a significantly greater proportion of switch events when compared to both the second COVID escalation period (*p* < 0.01), and COVID awareness period (*p* < 0.01). No significant differences were found when comparing the average number of monthly switches from three-month to one-month formulations between epochs. Privacy concerns prevented provincial level analysis to be conducted due to low cell size on this portion of the dataset.

## Discussion

Equal numbers of LAI new starts and discontinuations prior to and during the COVID-19 pandemic suggests this specific mental health service, for those already in care, continued throughout the pandemic. The consistent level of LAI discontinuation aligns with previous work showing that LAI use is often not maintained after initiation [17–19]; with LAI discontinuation often being a patient driven decision [17, 20]. There are a number of recognised barriers to LAI use, which may also contribute to discontinuation rates, including negative patient perceptions and/or limited awareness of LAI options, slower dose titration/adjustments, higher direct costs, and inconvenience of LAI therapy (e.g., logistical challenges related to dispensing/administration, and frequency of clinic visitations). There would be reason to suspect that during the COVID-19 pandemic many of the existing barriers to LAI use could have been exacerbated. In particular, the added challenges related to clinic visitations in the face of pandemic-related reductions in healthcare services. However, the observed consistency in rates of LAI new starts and discontinuations throughout 2019–2020 is indicative that clinics across the country were able to maintain services for this patient population – aligning with CPA and APA’s position that LAI treatment should be considered a medically necessary procedure during the pandemic [4, 5]. Interestingly, the continuation of LAI prescription rates contrasts with what has been published in other areas of healthcare, where new patient backlog volumes have grown steadily during the pandemic. For example, patient backlogs reached 13% for diabetes related illness, and 25% for cardiovascular diseases, resulting in dramatic decreases in medication dispensing as recently as March 2021 [21, 22].

Broad use of public insurance coverage for payment suggests lower SES in this patient population overall. Therefore, evidence of continuation of care for this group during the pandemic is important. In Canada, prescription payer type can be seen as a surrogate for SES. Canada has near universal healthcare coverage for services, but prescription drug coverage by public money varies by province with most provinces keying coverage to household income level [23, 24]. As a result, most individuals who qualify for pharmacare coverage in Canada are economically disadvantaged. The observation that most LAI Rx were filled and paid by publicly funded insurance plans suggests that the majority of individuals on LAIs were in a lower SES bracket, and that their access to medication by this route continued during the pandemic. However, the high numbers of individuals discontinuing LAIs could also be related to the public payer coverage, as many provinces resist covering LAIs as a first-line treatment, particularly SG-LAIs due to their comparatively high list price over oral antipsychotics [20, 25]. The data used for this study does not parse this out as there were roughly equal numbers of public payer individuals initiating and discontinuing, we however, did not examine the coverage limits of individual provincial drug insurance plans. Although it is difficult to draw parallels between healthcare systems, recent preliminary data from the United States suggests that the type of drug coverage patients’ have access to can impact overall LAI medication adherence. Comparing four-months of early pandemic data (March–June 2020) to four-months of pre-pandemic data (November 2019 – March 2020), LAI antipsychotic use declined by 1.6% for individuals insured commercially, 3.0% when insured through Medicade, and 5.3% for patients with Medicare [26].

Concomitant medication information showing significant use of valproate and lamotrigine in conjunction with LAIs suggests more common use of LAIs for several psychiatric conditions including bipolar disorder [27], and possibly borderline personality disorder [28, 29] than were expected. There is evidence from other parts of Canada that LAI antipsychotics are being prescribed for a number of mental health conditions that they are not approved for, as the only approval to-date is schizophrenia, schizoaffective disorder and bipolar disorder [30]. We did not have access to diagnosis data to examine what percentage of these prescriptions were for psychotic disorders as compared to other mental health disorders. However, in addition to efficacy concerns, there are also safety concerns with off label use of antipsychotics, so this is an area that should be further investigated [31–33].

When use of particular antipsychotics was examined, as expected SGAs were the predominate class prescribed. This is in line with other studies around the globe including in Sweden [34], Germany [35], and Spain [36]. The use of particular oral SGAs were similar to results seen in a study examining LAI use in a population of people living with schizophrenia in Germany, paliperidone was the most frequently prescribed LAI [35], however, in other studies risperidone microspheres are the most common LAI prescribed [36] which was the least commonly prescribed in our cohort.

Switches from one-month to three-month formulations significantly increased during the COVID-19 pandemic. This suggests that the convenience of the three-month formulation was well suited to the reduced in person clinical arrangements during the pandemic. The primary COVID-19 escalation period saw the greatest proportion of switches to the three-month formulation, but it was not significantly different from the pre-COVID-19 comparator period – the same monthly interval in the year prior to the pandemic. The significant decrease between the COVID-19 first escalation period to second COVID-19 escalation period could suggest that the majority of individuals who were interested in, or appropriate for switching from the one-month to the three-month formulations had already been switched, or there was greater access to clinics in the second escalation period reducing the need for longer-term injections. Our data does not provide sufficient detail to examine this question.

Despite ample evidence of the beneficial outcomes associated with LAI use, particularly in schizophrenia and psychotic disorders, and recommendations to expand use, the number of observed monthly LAI Rx new starts were consistently similar to the number of monthly LAI Rx discontinuations. While this pharmacy dataset does not cover all dispensing, these numbers show results similar to previous studies and suggest no further increases in LAI use in Canada over the period of study [37]. Alternatively, another interpretation for this pattern of use is that it could be reflective of off-label use of LAIs that is not leading to efficacious treatment responses, leading to individuals discontinuing the treatment such as has been reported by others [38]. Both of these potential reasons should be further investigated.

One further consideration should be mentioned with regard to antipsychotic use and COVID-19. There have been mixed reports of the impact of COVID-19 infection on individuals with severe and persistent mental illness [39–42]. In addition to the clear impetus to continue mental health services for those in need during the pandemic, there is also evidence that antipsychotics can affect coronavirus infection and may help prevent severe disease [43]. A recent report examined LAI antipsychotic treatment in a Spanish population and found that in this small cohort (*n* = 698), there was a lower prevalence of infection and a lower rate of severe outcomes [44]. Though, it is not clear whether the observed improvements were related pharmacologically to the use of antipsychotic medication, or whether patients treated with LAIs may be better able to follow appropriate public health guidelines. Considered together, this data highlights the importance of continuing mental health services for individuals with psychotic disorders during this pandemic to both prevent poor mental health outcomes, and to possibly mitigate death and disease from COVID-19 for this vulnerable population.

## Limitations

Data are restricted to Canada and may not reflect outcomes in other more severely affected countries. Data used for this study were generated from a privately managed dataset. This dataset did not contain diagnosis information and did not track travelling patients between pharmacies - though testing the influence of travelling patients on this dataset showed it had low impact on the results presented here. Furthermore, the dataset did not include any sociodemographic or psychopathological information, therefore we do not know if the included data are subject to selection bias.

## Conclusions

LAI prescriptions continued in a consistent pattern prior to, and during the COVID-19 pandemic in Canada. The consistent prescribing pattern of LAIs contrasts with other areas of healthcare, such as cardiovascular and diabetes care, which experienced decreases in medication prescribing during the COVID-19 pandemic.

## Supplementary Information

**Additional file 1.**

**Additional file 2.**

## Data Availability

The aggregate data supporting the conclusions of this study were obtained under license from IQVIA Real World solutions Canada utilizing their IQVIA Longitudinal Prescription (LRx) Database - January 2019 to December 2020 – and so are not publicly available. Aggregate data are however available from the authors upon reasonable request and with permission of IQVIA Real World Solutions Canada. Additional data can be obtained directly from IQVIA.

## Abbreviations

LAI: Long-Acting Injectable antipsychotic
SG-LAI: Second Generating Long-Acting Injectable Antipsychotic
FG-LAI: First Generation Long-Acting Injectable Antipsychotic
SGA: Second Generation Antipsychotic
FGA: First Generation Antipsychotic
COVID-19: Severe acute respiratory syndrome coronavirus 2 [SARS-CoV-2]
CPA: Canadian Psychiatric Association
APA: American Psychiatric Association
SES: Socioeconomic Status
LRx: Longitudinal Prescription
LAI Rx: Long-Acting Injectable Prescription
PP1: One-month paliperidone palmitate
PP3: Three-month paliperidone palmitate
