# Supplementary material for: Long-acting injectable antipsychotic (LAI) prescribing trends during COVID-19 restrictions in Canada: a retrospective observational study
Source: BMC Psychiatry. 2021 Dec 20;21:633. doi: 10.1186/s12888-021-03646-9 (PMC8687150; doi:10.1186/s12888-021-03646-9)
Supplement: Supplementary file 1 — Additional file 1. [file 12888_2021_3646_MOESM1_ESM.docx]

| **Epoch A** | **Epoch B** | **New Starts** | | | **Discontinuations** | | |
| --- | --- | --- | --- | --- | --- | --- | --- |
|  |  | **Epoch A, N (%)** | **Epoch B, N (%)** | **P-value** | **Epoch A, N (%)** | **Epoch B, N (%)** | **P-value** |
| Pre-COVID Comparator Period (Mar - May 2019) | Primary COVID Escalation Period (Mar - May 2020) | 1461.67 (25.95%) | 1534.33 (26.55%) | 0.47 | 1477.33 (26.23%) | 1525.67 (26.40%) | 0.84 |
| Pre-COVID Comparator Period (Mar - May 2019) | Second COVID Escalation Period (Sept - Nov 2020) | 1461.67 (25.95%) | 1509.67 (25.35%) | 0.46 | 1477.33 (26.23%) | 1530.67 (25.70%) | 0.52 |
| Primary COVID Escalation Period (Mar - May 2020) | COVID Maintenance Period (Jun - Aug 2020) | 1534.33 (26.55%) | 1529.33 (26.28%) | 0.74 | 1525.67 (26.40%) | 1494.00 (25.67%) | 0.37 |
| COVID Maintenance Period (Jun - Aug 2020) | Second COVID Escalation Period (Sept - Nov 2020) | 1529.33 (26.28%) | 1509.67 (25.35%) | 0.25 | 1494.00 (25.67%) | 1530.67 (25.70%) | 0.97 |
| Primary COVID Escalation Period (Mar - May 2020) | Second COVID Escalation Period (Sept - Nov 2020) | 1534.33 (26.55%) | 1509.67 (25.35%) | 0.14 | 1525.67 (26.40%) | 1530.67 (25.70%) | 0.39 |
| Pre-COVID Comparator Period (Mar - May 2019) | COVID Awareness Period (Dec 2019 - Feb 2020) | 1461.67 (25.95%) | 1519.33 (27.00%) | 0.21 | 1477.33 (26.23%) | 1446.67 (25.70%) | 0.52 |

**Supplementary Table 1**. Chi-square results comparing the average monthly proportion of LAI new starts and discontinuations between study epochs. Reported as total number of events (N) and percentage (%).
