# Supplementary material for: Long-acting injectable antipsychotic (LAI) prescribing trends during COVID-19 restrictions in Canada: a retrospective observational study
Source: BMC Psychiatry. 2021 Dec 20;21:633. doi: 10.1186/s12888-021-03646-9 (PMC8687150; doi:10.1186/s12888-021-03646-9)
Supplement: Supplementary file 2 — Additional file 2. [file 12888_2021_3646_MOESM2_ESM.docx]

**Supplementary Table 2**. Chi-square results comparing the average monthly proportion of one-month paliperidone palmitate (PP1) to three-month paliperidone palmitate (PP3) switches between study epochs. Reported as total number of events (N) and percentage (%).

| **Epoch A** | **Epoch B** | **PP1 to PP3 Switches** | | | **PP3 to PP1 Switches** | | |
| --- | --- | --- | --- | --- | --- | --- | --- |
|  |  | **Epoch A, N (%)** | **Epoch B, N (%)** | **P-value** | **Epoch A, N (%)** | **Epoch B, N (%)** | **P-value** |
| Pre-COVID Comparator Period (Mar - May 2019) | Primary COVID Escalation Period (Mar - May 2020) | 147.33 (4.26%) | 140.00 (4.02%) | 0.62 | 18.33 (2.12%) | 16.00 (1.36%) | 0.19 |
| Pre-COVID Comparator Period (Mar - May 2019) | Second COVID Escalation Period (Sept - Nov 2020) | 147.33 (4.26%) | 100.33 (2.82%) | **<0.01** | 18.33 (2.12%) | 19.67 (1.54%) | 0.32 |
| Primary COVID Escalation Period (Mar - May 2020) | COVID Maintenance Period (Jun - Aug 2020) | 140.00 (4.02%) | 88.33 (2.53%) | **<0.01** | 16.00 (1.36%) | 18.67 (1.52%) | 0.74 |
| COVID Maintenance Period (Jun - Aug 2020) | Second COVID Escalation Period (Sept - Nov 2020) | 88.33 (2.53%) | 100.33 (2.82%) | 0.46 | 18.67 (1.52%) | 19.67 (1.54%) | 0.97 |
| Primary COVID Escalation Period (Mar - May 2020) | Second COVID Escalation Period (Sept - Nov 2020) | 140.00 (4.02%) | 100.33 (2.82%) | **<0.01** | 16.00 (1.36%) | 19.67 (1.54%) | 0.71 |
| Pre-COVID Comparator Period (Mar - May 2019) | COVID Awareness Period (Dec 2019 - Feb 2020) | 147.33 (4.26%) | 102.67 (3.03%) | **<0.01** | 18.33 (2.12%) | 21.33 (1.97%) | 0.82 |
